# Geographic distribution of EMS missions and patient conditions in suburban and rural Sweden: a retrospective observational study

**DOI:** 10.1186/s13049-026-01561-0

**Published:** 2026-01-24

**Authors:** David Summermatter, Hans Blomberg, Henrik Aldén, Björn Äng, Anneli Strömsöe

**Affiliations:** 1 School of Health and Welfare, Dalarna University, Falun, S-79188 Sweden; 2 Department of Prehospital Care, Region Dalarna, Falun, S-79129 Sweden; 3 University of Uppsala, Uppsala, S-75310 Sweden; 4 Regional Development Administration, Region Dalarna, Falun, S-79129 Sweden; 5 Center for Clinical Research Dalarna, Uppsala University, Falun, S-79182 Sweden; 6 Department of Neurobiology, Care Sciences and Society, Division of Physiotherapy Karolinska Institutet, Stockholm, S-17177 Sweden

**Keywords:** Emergency medical services, Telemedicine, Remote consultation, Urban health services, Patient care, Geographic information system

## Abstract

**Objective:**

Swedish emergency medical services (EMS) faces challenges due to increased demand and limited resources. In Sweden, EMS have experienced a general increase in workload, with longer response times, and limited access to and capacity of receiving facilities. The geographical distribution of EMS events remains unknown. The study aims to investigate the occurrence of EMS missions and assessment of patients' conditions by mapping and comparing the geographical distribution between a Swedish suburban and a rural setting.

**Methods:**

A descriptive observational study assessed patient conditions and geographical distributions of EMS events in a medium-sized region in Sweden in 2018. Data was retrieved from ambulance medical records.

**Results:**

EMS assessed 24,672 patients, of whom two-thirds had non-urgent conditions. Almost half of the patients were females, and the median age of all patients was 72 years. Suburban areas had a slightly higher proportion of urgent dispatch priority than rural areas, while patient contact times were considerably longer in rural areas. There were no notable differences in suburban versus rural areas regarding which conditions were most common. Overall, 29% of patients were left on-scene after EMS assessment. Rural units more often left patients on-scene compared with suburban units.

**Conclusions:**

Overall, the EMS events were broadly equal between suburban and rural areas. Despite longer transport and patient contact times in rural areas, response times were still similar. However, patients in rural areas are assessed and left on-site to a greater extent compared to patients in suburban areas, who are instead transported to hospitals for discharge.

**Trial registration:**

Not applicable.

**Supplementary Information:**

The online version contains supplementary material available at 10.1186/s13049-026-01561-0.

## Background

Swedish prehospital care faces several challenges, including increased demand for emergency medical services (EMS), overcrowding in emergency departments, an aging population with multimorbidity, and the need for improved care for specific patient groups such as geriatric trauma patients [1–3].

Recently, EMS in Sweden experienced a general increase in workload with longer response times, and limited access to and capacity of receiving facilities [1]. In some parts of northern Europe and North America, a significant proportion of ambulance activations (16—51%) involve patients assessed and released on scene, without being transported or treated [4–6]. Previous studies have shown that patients initially assessed by EMS were divided into three different groups based on EMS assessment: patients unlikely to need an emergency department (ED) visit, patients in need of evaluation before an ED referral, and patients likely needing an ED evaluation [7, 8]. This development creates challenges regarding response times, resource allocation and the efficiency of the ambulance system and requires assessment of patients who are unlikely to require an emergency department visit [9, 10].

These three distinct patient groups have unique needs. Regrettably, there is a considerable lack of understanding of how ambulance activations are distributed geographically, particularly between suburban and rural areas. In addition, little is known about the factors that determine whether a patient’s condition necessitates transportation to a health care facility or if they can be referred to another level of care. The interplay of geography, infrastructure, and ambulance preplanned locations due to population density impacts not only ambulance response times, but also resource allocation and the quality of care provided by EMS [11]. Sweden consists of both urban, suburban and rural populated areas, where the most populated areas are usually located along the coast and where the inland areas are more sparsely populated [12]. By using geographical tools such as Eurostat Degree of Urbanization (DEGUBRA), it might be useful to get knowledge of how the population is distributed across land area and its accessibility to healthcare [13].

To our knowledge, no previous study has mapped EMS events from a population density perspective using heat maps. The study aims to increase knowledge about the occurrence of EMS missions and the assessment of patients' conditions by mapping and comparing the geographical distribution between a Swedish suburban and a rural setting. A secondary aim was to identify possible differences in distance traveled to reach patient, response times, and patient contact times in suburban missions compared to rural missions.

## Methods

### Study design and setting

A retrospective observational study was performed using a quantitative approach. The study was conducted in Dalarna, a medium-sized region, situated in central Sweden. The region covers an area of 28,188.8 km^2^ with a population of 287,191 (2018). The population density in Dalarna was 10.2 inhabitants per km^2^ in 2018. Population data from 2018 were used to align with the year of data collection and ensure contextual relevance. In that year, there was a variation in population density from 1 to 89 inhabitants per km^2^ (2018) [12]. The municipalities in Dalarna that were included in this study are classified as suburban and rural areas, as described in the DEGUBRA and its methodological manual published by Eurostat to define cities, towns, and rural areas for international comparison [13].

The EMS organization is, as all Swedish EMS organizations, a tax-funded EMS system providing a 24/7, non-tiered, multipurpose, and advanced life support ambulance fleet staffed with emergency medical technicians and registered nurses (RNs).

RNs employed in EMS have at least three years of university studies, and most of those in the prehospital care workforce have an additional year of education with a postgraduate diploma in prehospital care, anesthetic care, or intensive care. Emergency medical technicians are trained as assistant nurses [14, 15]. In Dalarna, EMS nurses may, under protocol-driven collaboration, refer patients not requiring hospital transportation to municipal home care or primary health care follow-up. This study follows the Strengthening the Reporting of Observational Studies in Epidemiology (STROBE) guidelines [16].

### Data collection

The study sample included patients of all ages who were attended by EMS units during the year 2018. The inclusion criteria were primary emergency events with EMS units arriving, having contact with a patient and assessing their condition on-scene. Exclusion included EMS interfacility transports and instances where no patient was assessed, such as activations prior to arrival by the dispatch center and area coverage activations made by other EMS units in other municipalities. In addition, cases of refusal to assess, or very limited assessment, and cases of support service to other ambulance units or fire departments were also excluded. Furthermore, the contact times include both on-scene and transport times. The data were retrieved from digital ambulance medical records by independent collaborators in 2018, who were not involved in the study. The medical records were created by RNs in EMS. Variables that were collected included age, sex, location, clinical data, intervals, distance travelled, treatment, destination, and prehospital patient assessment.

### The Eurostat Degree of Urbanization (DEGUBRA)

The Eurostat DEGUBRA classification system was used to categorize EMS stations into suburban and rural areas [13]. Suburban areas are defined as having at least 300 inhabitants per km^2^ and a minimum population of 5,000, with less than 50% of the population in urban centres and no more than 50% in rural grids, according to Eurostat DEGURBA. In this study, there were five EMS stations operating in suburban areas. Rural areas are defined as having more than 50% of their population in rural grids and may contain smaller towns and villages. In this study, six EMS stations operated in rural areas, covering larger geographical areas with smaller populations [13].

### The Rapid Emergency Triage and Treatment System (RETTS) and ESS color

The Rapid Emergency Triage and Treatment System (RETTS) is a two-step triage system consisting of one algorithm for vital signs and one algorithm for emergency signs and symptoms [17]. The combination of the two algorithms generates a triage color on a five-level priority scale level describing the need for medical attention from physicians and nurses at an ED. The system encompasses the colors red, considered urgent, orange, considered potentially urgent, yellow, considered non-urgent, green considered non-urgent, and blue, indicating no need for emergency care. Patients identified as blue, that is, no need for emergency care, are therefore not included in the study sample. The RETTS is used to assess the urgency of a patient’s condition upon arrival on-scene, while the level of care needed (EMS treatment only, ED or primary care) is decided separately at dispatch or on-scene. Integration of the RETTS allows automated triage classification during initial patient assessment based on vital signs like respiratory rate, blood pressure, oxygen saturation, and heart rate. The RETTS is also used as a decision-making triage tool to determine the level of care needed and can guide EMS clinicians in referring patients to alternative care settings like primary care, self-care advice, or arrangement of own transportation to an ED [17, 18].

### Urgent and non-urgent conditions and assessed conditions

EMS clinicians assess patients on-scene, based on a structured assessment and management protocol, after which they perform a categorization using the RETTS system. The EMS assessment findings determine whether the condition is considered urgent (level red or orange), requiring immediate care and transport, or non-urgent (level yellow or green) [17], which may either be managed on scene or require transportation to a receiving health care facility such as ED or primary care. The category blue, indicating no need for emergency care is part of RETTS, but is not operationalized in Swedish EMS. For analysis, the EMS assessed conditions were divided into two categories: urgent (life-threatening) and non-urgent (non-life-threatening). The urgent category included the red and orange priority levels, indicating a need for immediate EMS assessment, treatment and urgent transport to a receiving facility for medical care. The non-urgent included yellow and green priority level, indicating a lower need for urgent EMS assessment and transport.

Grouping similar priority levels might provide insight into EMS operations and align with common EMS triage practices that consider patient needs and resource allocation.

### Dispatch priority and transport priority level

The views on levels of urgency vary between EMS organizations worldwide. In Sweden, in 2018, emergency dispatch centers communicated priority levels to EMS units in a system different from the above-described RETTS. In the dispatch system, priority 1 meant activation with lights and sirens and was used for life-threatening or critical conditions. Priority 2 meant urgent, but non-life-threatening conditions. Priority 3 meant low acuity activations where patients could wait some time for an EMS unit to arrive on-scene [19, 20]. EMS units in Dalarna occasionally performed priority 4 assignments, which involved scheduled transfers.

### Statistical analysis

Descriptive statistics in this study included frequencies and percentages for categorical variables. Continuous variables were shown as medians, quartiles, and minimum–maximum ranges. Non-parametric statistical methods were used that involved the Chi-squared test for categorical variables and the Mann–Whitney U-test and Kruskal–Wallis test for continuous variables. Binary logistic regression analyzed the potential association between the dependent variable (ESS color) and the independent variable (suburban vs. rural area), adjusting for age and sex. We also performed a linear regression with a log-transformed outcome to examine the possible relationship between the dependent variable (response time) and the independent variable (suburban vs. rural), adjusting for age and sex. A *p*-value of less than 0.05 was considered significant. Two-tailed tests were used. Data analysis was performed using IBM SPSS Statistics (version 26) [21].

### Maps

A combination of R (v4.3.2) [22] and Quantum Geographic Information System (QGIS) 2024 (version 3.28.8.) [23] was used for geocomputation, to generate a visual overview of EMS activations and, explore patterns related to EMS units and patient location. A geospatial indexing hexagonal grid system, Hexagonal Hierarchical Spatial Index (H3), was used to visualize population density [24]. Population data were aggregated to H3 hexagons for spatial analysis and visualization. Kernel density estimation in QGIS was applied to produce heat maps depicting the density of EMS scene locations and patients left on-site. The integration of population data from Dalarna provides insights into distribution and ambulance preplanned locations due to population density.

### Declaration of generative AI in scientific writing

We used OpenAI’s ChatGPT AI language model to optimize R codes during the geographical data cleaning process and to generate H3 [24] hexagons for the maps and all AI-suggested code was human-reviewed. However, visualization of the results, was done in QGIS [23] without any assistance of AI. The authors take full responsibility for the contents of this publication.

## Results

**Fig. 1:**
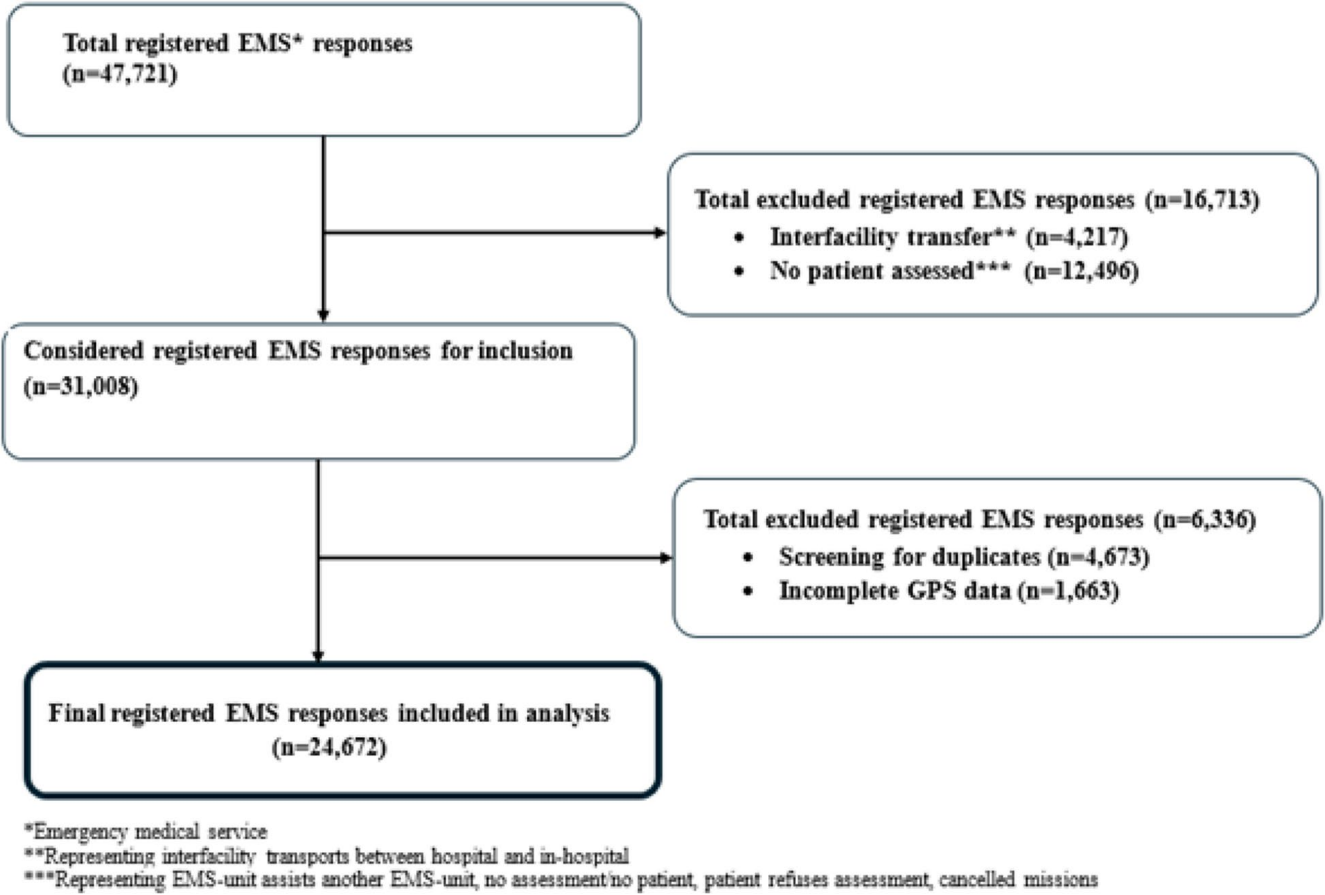
Flowchart of registered emergency medical service responses

In total, 24,672 EMS events from 2018 were evaluated. Each event was represented by one patient (Fig. 1).

### Demographics and patient characteristics

The median age was 72 years for patients in suburban areas and 70 years in rural areas (Table 1). Male patients constituted just over half of the patients assessed by EMS units (50.9%) and with a slightly higher proportion in rural areas (52.9%). Most of the dispatched patients in the suburban areas were classified as urgent (priority 1, 35% and priority 2, 59.2%), while rural areas reported a lower dispatch priority 1 (33.7%), but a marginally higher dispatch priority 2 level (60.3%). However, at the point of transport, after EMS assessment, rural areas reported a higher transport level of priority 1 (12.5%) compared to suburban areas (11.2%). In rural areas, there were slightly lower percentages transported with priority 3 and 4 (4.1% and 0.8% respectively) compared with suburban areas (6.2% and 0.9%). For patients left on-scene, transport priority level was not assigned and is reported as missing data (24.9%).

**Table 1:** Demographics and patient characteristics, EMS assessment for patients assessed on-scene by suburban and rural EMS units

	All EMS units	Suburban	Rural	
	N (%) Total = 24,672 (100)	N (%) Total = 18,698 (75.8)	N (%) Total = 5,975 (24.2)	*P*-value*
Sex				< 0.001
Male	12,451 (50.9)	9,333 (50.3)	3,118 (52.9)	
Missing	224 (0.9)	146 (0.8)	78 (1.3)	
Age years				< 0.001
Median (Q1, Q3)	72 (48, 82)	72 (48, 83)	70 (46, 81)	
Missing	173 (0.7)	116 (0.6)	57 (1)	
Dispatch priority level^1^				< 0.002
1	8,564 (34.4)	6,552 (35.0)	2,012 (33.7)	
2	14,673 (59.5)	11,072 (59.2)	3,601 (60.3)	
3	1,323 (5.4)	975 (5.2)	348 (5.8)	
4	113 (0.5)	99 (0.5)	14 (0.2)	
Transport priority level^2^				< 0.001
1	2,845 (11.5)	2,099 (11.2)	746 (12.5)	
2	14,080 (57.1)	11,137 (59.6)	2,943 (49.3)	
3	1,392 (5.6)	1,150 (6.2)	242 (4.1)	
4	211 (0.9)	164 (0.9)	47 (0.8)	
Missing	6,145 (24.9)	4,148 (22.2)	1,997 (33.4)	
ESS color^3^				< 0.001
Vital signs only	527 (2.1)	389 (2.1)	138 (2.3)	
Red	798 (3.2)	602 (3.2)	196 (3.3)	
Orange	6,522 (26.4)	4,941 (26.4)	1,581 (26.5)	
Yellow	9,712 (39.4)	7,506 (40.1)	2,206 (36.9)	
Green	6,289 (25.5)	4,629 (24.8)	1,660 (27.8)	
Missing	825 (3.3)	631 (3.4)	194 (3.2)	
Destination^4^				< 0.001
Hospital	17,626 (71.4)	14,215 (76.0)	3,411 (57.1)	
Left on-scene	6,235 (25.3)	4,204 (22.5)	2,031 (34.0)	
Health care centre	636 (2.6)	178 (1.0)	458 (7.7)	
Other	115 (0.5)	63 (0.3)	52 (0.9)	
Missing	61 (0.2)	38 (0.2)	23 (0.4)	
Top 10 conditions assessed on-scene by EMS^5^				
Chest/thoracic pain	2,602 (10.5)	1,935 (10.3)	667 (11.2)	
Dyspnoea	2,217 (9.0)	1,749 (9.4)	468 (7.8)	
Abdominal/flank pain	1,819 (7.4)	1,385 (7.4)	434 (7.3)	
Fever	1,293 (5.2)	986 (5.2)	325 (5.4)	
Trauma/head injury	1,190 (4.8)	930 (5.0)	260 (4.4)	
Non-specific condition	931 (3.8)	784 (4.2)	147 (2.5)	
Injury hip/thigh bone	778 (3.2)	596 (3.2)	182 (3.0)	
Dizziness	631 (2.6)	498 (2.7)	133 (2.2)	
Intoxication	541 (2.2)	446 (2.4)	95 (1.6)	
Seizure disorder	536 2.2)	433 (2.3)	103 (1.7)	

*EMS* Emergency Medical Services

^*^significant < 0.05

^1^ *Dispatch priority level* refers to the urgency determined by the emergency medical dispatch center at the time of the activation

^2^ *Transport priority level* is the EMS clinician’s clinical decision on the urgency of transport to a medical facility

^3^ *ESS* = Emergency Symptoms and Signs. *ESS color* refers to the Emergency Signs and Symptoms triage system, where red = urgent, orange = potentially urgent, yellow = non-urgent, green = non-urgent. The variable *vital signs only* indicates that EMS assessed the patient solely by documentation of vital signs without assigning an ESS color

^4^ *Destination* denotes the location to which the patient was taken or left (e.g., hospital, on-scene discharge)

^5^ *Conditions assessed on scene* are based on EMS clinical assessments and documentation at the scene

In total, according to ESS color after EMS assessment, one-third of the patients (29.6%) were assessed to be in urgent need of care (red or orange ESS color), and there was a slight difference in assessed ESS color between suburban versus rural areas (29.6% vs 29.8%, *p* < 0.001). Adjusting for age and sex did not change the outcome. However, there was a notable difference between suburban and rural areas regarding patient destination. In rural areas, patients were left on-scene more often compared to suburban areas (34% versus 22.5%) and visited health care centers more often (7.7% versus 1.0%), whereas suburban patients were more often transported to hospital 76.0% versus 57.1%. The condition most commonly assessed by EMS units in suburban and rural areas combined was chest/thoracic pain (10.5%), closely followed by dyspnea (9.0%). There were slightly higher proportions of dyspnea (9.4% versus 7.8%), abdominal/flank pain (7.4% versus 7.1%), and trauma (5.0% versus 4.4%) were more common in suburban areas than in rural areas.

### Characteristics and distribution of patient contact times (Table 2)

**Table 2:**
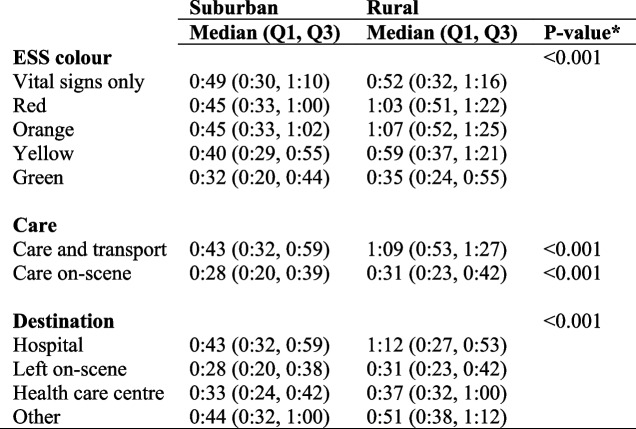
Characteristics and distribution of patient contact times for EMS in suburban and rural areas. Presented as hours and minutes (h:mm)

*EMS *Emergency Medical Services

*ESS* Emergency Symptoms and Signs

*significant <0.05

There was a difference in patient contact time between suburban and rural areas, with longer contact times in rural areas, especially for patients with urgent conditions. The patient contact times for ESS color red was 1:03 versus 0:45 and orange 1:07 versus 0:45, respectively. The overall patient contact time for care and transport was longer in rural areas than in suburban areas (1:09 versus 0:43). However, contact time was longer for patients in rural areas who had a hospital as their destination and shortest when the patient was left on site in suburban areas (0:28 versus 0:31).

### Distance and time of transport and on-scene (Table 3)

**Table 3:**
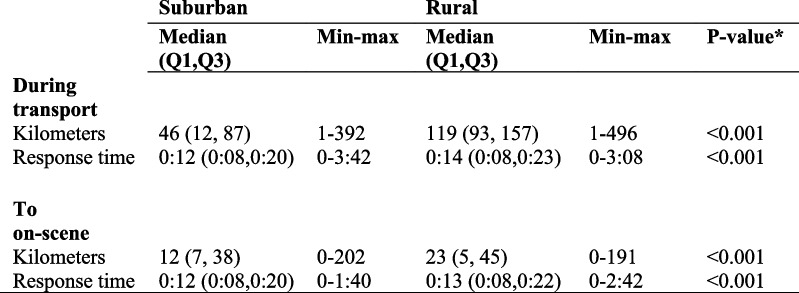
Distance and time during transport and to the scene in suburban versus rural areas

*significant <0.05

The distances from the point of dispatch to arrival were longer for EMS units in rural areas (23 km) than in suburban areas (12 km). Nevertheless, the response time from dispatch to arrival was only slightly longer in rural versus suburban areas (0:13 vs 0:12, *p* < 0.001). Adjusting for age and sex did not change the outcome.

### EMS activation (Fig. 2)

**Fig. 2:**
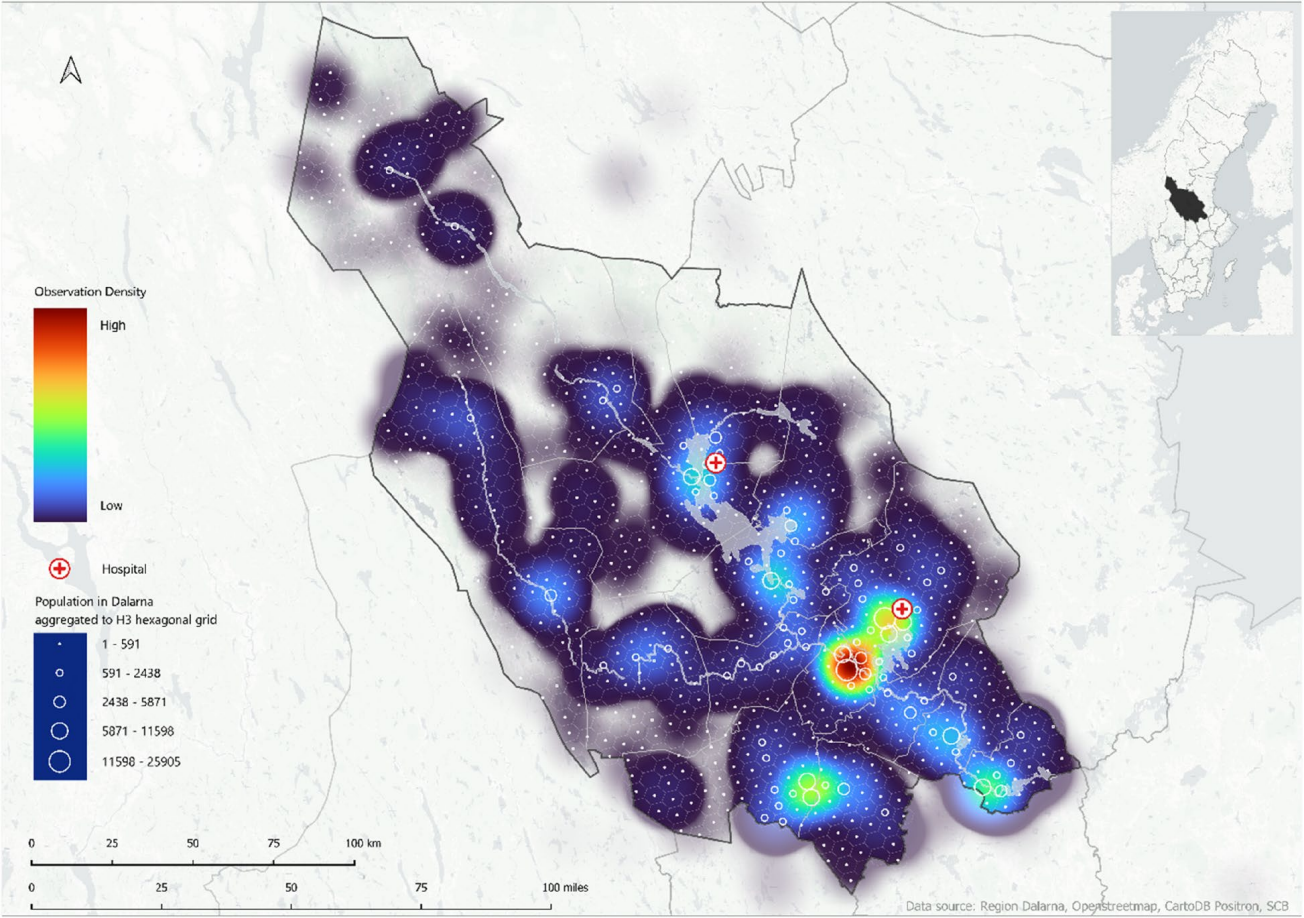
footnotes: Visualization of EMS scene location for all EMS activations during 2018 in Dalarna, from low (blue) to high observation density (red). Heat map generated using Kernel density estimation and population densities are represented by H3 hexagonal grid system and ranging from 0 to 25,905. Hospitals are indicated with red crosses

The EMS activations were visualized in heat maps where red and orange represented high density of activation, and light and dark blue indicated areas with low density of activation. Higher activation frequencies were observed in suburban areas, whereas lower activation frequencies were common in sparsely populated areas. The heat maps revealed clear spatial patterns of EMS activations across Dalarna. The first map, illustrating all EMS missions during 2018, showed clusters around the major population centers of Falun and Borlänge, as well as in the southern parts of the region near Ludvika and Avesta. In contrast, the northern municipalities exhibited sparse EMS activities, corresponding to less densely populated areas. Smaller clusters were also observed around Mora, Leksand, Vansbro and Malung.

### Care provided on-scene only (Fig. 3)

**Fig. 3:**
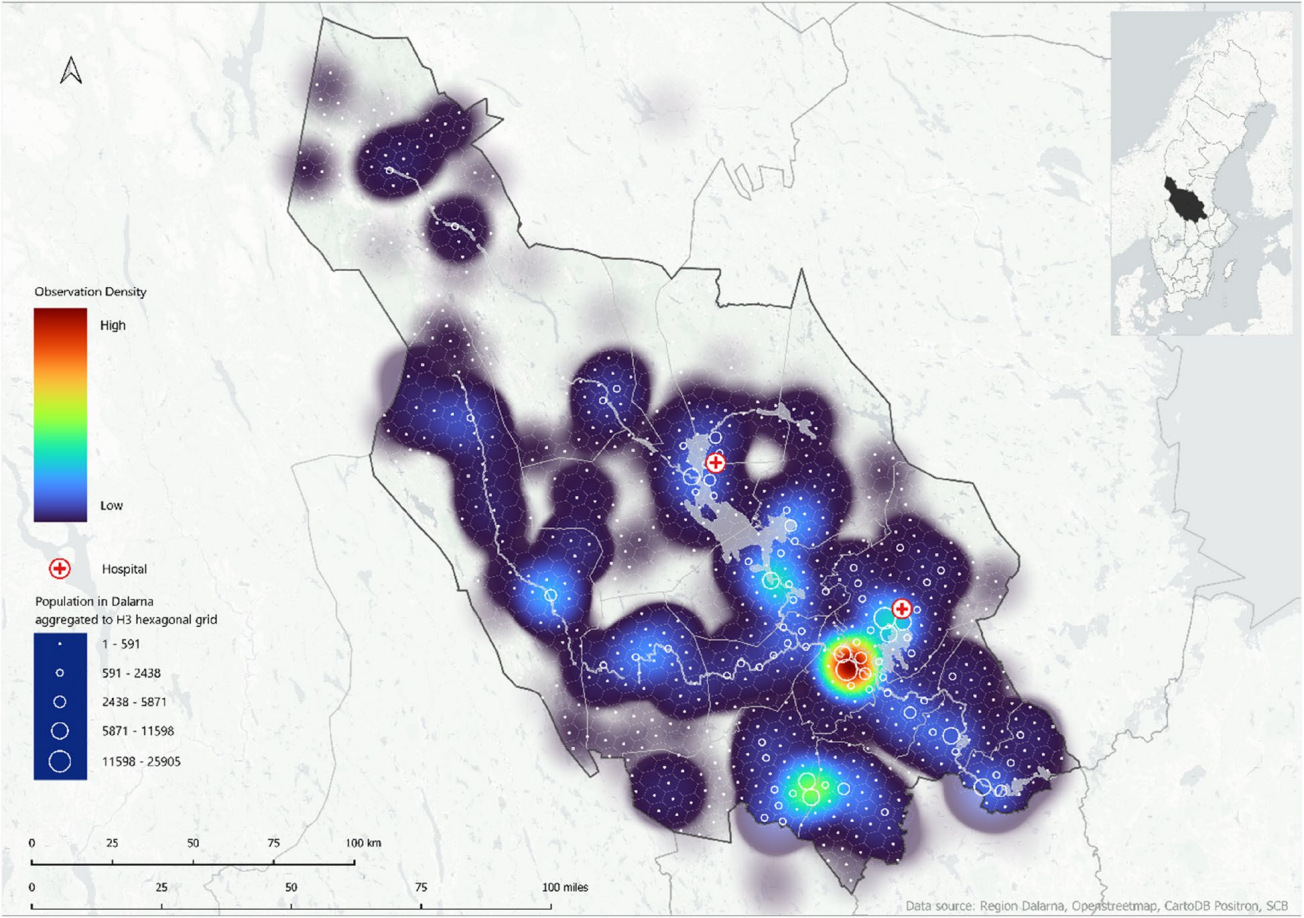
footnotes: Heat map visualizing the locations of all patients discharged on-scene by EMS in Dalarna during 2018. This map was generated using Kernel density estimation and population densities are represented through the H3 hexagonal grid system and ranging from 0 to 25,905. Color-coded from low observation density (blue) to high observation density (red). Hospitals are indicated with red crosses

The spatial distribution of patients left on-scene in suburban and rural areas was visualized in relation to population density. The second map, illustrating these patients, revealed a similar clustering pattern, with high densities around Falun and Borlänge, and smaller clusters appearing around Mora, Leksand, Vansbro, and Malung.

## Discussion

The main findings of this study were that patient conditions did not differ significantly between suburban and rural EMS missions, but rural missions involved longer patient contact and transport times. In addition, most patients were dispatched as urgent, a finding that was stronger in suburban areas than in rural areas. In rural areas, more patients were left on–scene than in suburban areas, whereas patients in suburban areas were more likely to be left in hospital. Another important finding was that the patient contact time was longer in rural areas than in suburban areas. This time was the shortest when a patient was left on-scene. The response time from dispatch to arrival was barely longer in rural areas than in suburban areas. Similar findings have been reported by Alruwaili and Alazany in 2022 [25]. This recent systematic literature review of 37 studies showed that EMS responses, on-scene and transport times, are generally longer in rural areas than in urban areas. This underscores the challenges of EMS in rural settings.

In this study, data were described and analyzed using the Eurostat DEGUBRA classification system [13]. The studied region had a population density that could only fulfil the criteria for suburban and rural populated areas. Less than half of the EMS stations were found in suburban areas, with the remainder in rural areas. Overall, the distribution of female and male patients was roughly equal. The EMS units were most often activated for an older adult patient population, with a median age of 72 years for suburban areas and 70 years for rural areas. Our findings are in line with those of other EMS studies [26]. Another study focusing on trauma in older adults highlighted challenges for EMS to care for such patients [2]. This might reflect an increased need for health care interventions with a scope of practice beyond the traditional EMS service serving suburban and rural areas [27].

Most of the suburban and rural assignments assessed by EMS were coded as yellow, indicating non-urgency, with limited waiting time for the patients, or orange, indicating urgent, potentially life-threatening conditions. These results are consistent with previous data [28].

However, the majority of cases in rural areas were classified as yellow or green, whereas most suburban cases were orange or yellow. This may suggest differences in types of patients seen in rural areas, although the underlying causes cannot be determined by our data. In rural and remote areas, EMS units often operate with an expanded scope of practice and may provide treatment on-scene, potentially avoiding the need for transportation to hospital [27]. The fact that patients are often transferred to hospitals in suburban areas may be due to the assumption that ambulance personnel do not have direct contact with health centers and, therefore, are unable to book appointments for patients. This might make them more likely to transfer a patient to an ED [29]. A systematic review is currently underway to investigate models of collaboration between EMS and primary care [30]. In our study, the transport priority level was used as a proxy to categorize cases as urgent or non-urgent. Most of the patients in this study were transported as urgent. The RETTS provides a valuable tool for triage but is not without limitations. Triage decisions are subjective; signs and symptoms can be interpreted differently, which can result in variation of color assignments [31]. The variable “vital signs only” indicates that EMS assessed a patient only by documentation of vital signs without an ESS code, which is required to generate a triage color. In our data, patients who were allocated to this category often had cardiac arrest. However, the initial ESS color was determined at least twice in each case and was not always consistent with the final ESS color. In our study, the most common symptoms were chest/thoracic pain, dyspnea, and abdominal/flank pain. This is in line with earlier results [32]. Suburban EMS units encountered fewer patients with chest/thoracic pain than rural EMS units. However, suburban units had a higher incidence of dyspnea, suggesting that patients living in suburban areas may experience more respiratory-related emergencies. This requires further investigation into environmental or lifestyle differences. A previous study has shown that remote visual assessment of medical conditions such as chest pain, shortness of breath, and abdominal pain, categorized as non-urgent, could play an important role in diagnosis and triage decisions [33]. Digital technology using remote visual assessment, either between dispatch and patient, or on-scene EMS and physician, would add a valuable layer and support EMS in clinical decision-making and may reduce unnecessary hospital transport. Such approaches might help to address disparities in rural areas by supporting EMS clinicians when immediate access to health care facilities is limited. The majority of EMS unit dispatches were urgent in both suburban and rural areas. The percentage of suburban EMS unit activations that were dispatched for priority 1was higher than for rural EMS units. This could be explained by demographics and differences in population density between suburban and rural areas, and optimization of EMS resource use based on dispatch center guidelines. The results are consistent with those of previous studies [11, 34]. In our study, most transports were coded with triage level 2 (57.1%), which means urgent but non-life-threatening conditions. In rural areas, EMS units had a slightly higher proportion of priority 1 transports than in suburban areas, a pattern that was also observed for trauma patients by Waalwijk et al. in 2022 [34]. The priority level by the EMS staff for transporting a patient to a healthcare facility can be influenced by a number of factors, such as the severity of the patient's condition and available ambulance resources. A previous study shows that mortality was not associated with the transport time from dispatch to arrival at the hospital [35]. However, in this study, the mortality was not one of the outcomes but in retrospect, it can be concluded that it has been interesting to study mortality in relation to the study's data on response and transport times. Further, the emergency dispatcher assessed two-thirds of the calls as urgent, while the EMS staff assessed that one-quarter could be left at the scene and did not require transport to the hospital. Overall, more patients were left at the scene in rural areas than in suburban areas, which is consistent with previous research [20]. There is an increasing trend toward treating non-urgent patients at home and our findings may underscore the need for future strategies for EMS to provide patient home care. Relevant to our findings is whether care in the future will take place in homes instead of in hospitals. Adaptive health care technology is on the rise and can be a resource for patients living in rural areas and in need of care [36]. The distance to the nearest hospital and the level of competence at EMS units play crucial roles and influence the decision-making process regarding transport priority [37]. Longer transport times from rural areas to health care facilities can affect the assessment of severity and need for emergency medical care. In our study, rural areas had a higher proportion of transport level priority 1, and the relationship between distance and health outcomes should be considered and cannot be dismissed [38].

Patient contact times varied between suburban and rural areas. The patient contact times were longer in rural areas than in suburban areas, and care levels during transport were. In addition, more time was spent with patients in rural areas who were left in hospital after transport [25, 39]. Furthermore, there was a difference between suburban and rural areas in distance travelled during patient assignments. The transportation distance was twice as long in rural areas compared to than in suburban areas, but the response time did not differ. As regards time to the scene, there was barely any difference between suburban and rural areas. This suggests that the transport times are longer for patients living in rural areas, but the response times appear to be quite similar to suburban areas [25]. These findings indicate that EMS resources may be located equidistantly from the population in suburban and rural areas, and thus, the patients will initially receive an equal assessment of health care needs.

### Limitations

A strength of the study is the low level of missing data, which improved the quality of the presented heat maps and tables. This also yielded valuable insights that can contribute to the identification of challenges in each specific context and enable a more comprehensive understanding of EMS events in a region with suburban and rural areas. However, there are limitations in this study. A proxy was needed for data related to the triage level for ambulance transport, which reduced the quality of the data. The reported initial RETTS color may not always reflect the true severity of patient conditions, which could have affected results. Therefore, the decision was made to report only the initial priority level and to use transport priority as a proxy for the level of urgency. Although Dalarna encompasses both suburban and rural areas, its geographical characteristics might limit the generalizability of the findings. The region encompasses 6.3% of Sweden’s land area, but only 2.7% of its population [12]. In addition, the data were collected during a single year (2018), and seasonal variations in EMS activations may have influenced the results.

## Conclusions

Overall, there were no major differences in the EMS events that occurred in suburban or rural areas. As expected, there were longer transport and contact times with patients in rural areas than in suburban areas. Remarkably, the response times were similar regardless of where an EMS event occurred. However, patients in rural areas are assessed and left on-site to a greater extent compared to patients in suburban areas, who are instead transported to hospitals for discharge.

## Supplementary Information

Supplementary Material 1.

## Data Availability

The dataset generated for the current study are not publicly available due to privacy regulations but is available from the corresponding author on request with appropriate ethical approval.

## Abbreviations

EMS: Emergency Medical Services
ED: Emergency Department
RETTS: Rapid Emergency Triage and Treatment System
ESS: Emergency Signs and Symptoms
RN: Registered Nurse
QGIS: Quantum Geographic Information System
H3: Hexagonal Hierarchical Spatial Index
SPSS: Statistical Package for the Social Sciences
